# Remarkable Memories

**Published:** 1889-01

**Authors:** 


### REMARKABLE MEMORIES.

That the human memory may be largely developed and improved by certain methods of discipline is an accepted fact, but the ability of retention that some minds have cannot be explained in such way. It can only be called phenomenal. There was a Corsican boy who could rehearse forty thousand words, whether sense or nonsense, as they were dictated, and then repeat them in a reversed order, without making a single mistake. A physician about sixty years ago could repeat the whole of "Paradise Lost" without a mistake, although he had not read it for twenty years. Euclid, the great mathematican, when he became blind, could repeat the whole of Virgil's "Æneid," and could remember the first line and the last line in every page of the particular edition which he had been accustomed to read before he became blind. One kind of retentive memory may be considered as the result of sheer work, a determination toward one particular achievement, without reference either to cultivation or to memory on other subjects. This is frequently shown by persons in humble life in regard to the Bible. An old beggarman at Sterling, known about fifty years ago as "Blind Alick," afforded an instance of this. He knew the whole of the Bible by heart, insomuch that if a sentence was read to him, he could name the book, chapter and verse; or if the book, chapter and verse were named, he could give the exact words. A gentleman, to test him, repeated a verse, purposely making one verbal inaccuracy. Alick hesitated named the place where the passage was to be found, but at the same time pointed out the verbal error. The same gentleman asked him to repeat the ninetieth verse of the seventh chapter of the Book of Numbers. Alick almost instantly replied: "There is no such verse. That chapter has only eighty-nine verses." Gassendi had acquired by heart six thousand Latin verses; and in order to give his memory exercise, he was in the habit of daily reciting six hundred verses from the different languages.

